# Role of Circular RNAs in Liver Diseases

**DOI:** 10.7150/ijms.124473

**Published:** 2026-01-01

**Authors:** Badr Alzahrani

**Affiliations:** Department of Clinical Laboratory Sciences, College of Applied Medical Sciences, P.O. Box 2014, Jouf University, Aljouf Province, Saudi Arabia.

**Keywords:** circular RNA, liver, NAFLD, liver fibrosis, HCC

## Abstract

Circular RNAs (circRNAs) are a subclass of noncoding RNAs characterized by their closed-loop structure without terminal 3′ or 5′ ends. Studies have shown that circRNAs play pivotal roles in the regulation of various cellular processes. These molecules function as microRNA (miRNA) sponges, interact with RNA-binding proteins, and modulate gene transcription. CircRNAs are vital for regulating liver homeostasis, and dysregulation of their expression is correlated with liver diseases such as hepatic fibrosis, steatosis, inflammation, and liver cancer. Elucidating the functional significance of circRNAs in liver diseases is crucial, as this knowledge may facilitate the identification of novel diagnostic biomarkers and therapeutic targets for conditions that contribute significantly to global morbidity and mortality. This review aimed to highlight current research underscoring the functional roles of circRNAs in the molecular pathogenesis and progression of liver diseases, including hepatocellular carcinoma, nonalcoholic fatty liver disease, and liver fibrosis. To provide an updated and comprehensive overview, a literature search was conducted across major scientific databases. This review reveals that circRNAs perform multifaceted functions in liver homeostasis and disease by regulating gene expression through miRNA sponging, interacting with signaling pathways, and influencing cellular processes, including vascularization, metastasis, the cell cycle, apoptosis, cellular stress, metabolic activity, inflammatory responses, and cellular senescence. Despite their pivotal involvement in liver diseases, translating circRNA-based research into clinical practice remains challenging. In conclusion, circRNAs represent an emerging frontier in liver disease research, offering considerable promise for future clinical applications.

## Introduction

Circular RNAs (circRNAs) represent a distinct subclass of noncoding RNAs that exist in a covalently closed-loop form. Their closed-loop structure, devoid of enzyme recognition elements, confers resistance to exonuclease degradation; thus, circRNAs exhibit greater stability and longer half-lives than linear RNAs 1. Although predominantly located in the cytoplasm, circRNAs are also present in the nucleus; however, the mechanisms governing their export from the nucleus remain poorly understood. CircRNAs were first discovered in plant viroids in 1976 and later in the hepatitis delta virus. Two principal models have been proposed to explain circRNA biogenesis: exon skipping (lariat) and back splicing 2. Based on their biogenesis, several types of circRNAs have been identified, including sense overlapping, antisense, intronic, exonic, and intergenic circRNAs 3.

CircRNAs affect the expression of many mammalian genes both transcriptionally and post-transcriptionally through transcriptional regulation, collaboration with microRNAs (miRNAs), and splicing interference. During transcription, nuclear circRNAs interact with RNA polymerase II and the U1 small nuclear ribonucleoprotein particle complex 4. A subset of circRNAs is produced through the back-splicing of pre-mRNAs to generate circular transcripts. CircRNAs serve as binding sites for miRNAs and RNA-binding proteins, facilitating their role in post-transcriptional gene regulation. In addition, circRNAs can inhibit linear RNA splicing and regulate their expression. For example, *Drosophila* muscle-blind circRNAs drive their expression through the alternative splicing of precursor RNA 2.

Although circRNAs are considered non-coding RNAs, they can also encode proteins. For example, the groundbreaking research by van Heesch et al. on 80 human heart translatomes identified at least 40 circRNAs that encode proteins 5. Another example is the hepatitis delta virus circRNA, which translates into a 122-amino acid protein within infected cells. In protein-coding circRNAs, cap-independent translation occurs via internal ribosome entry sites, N6-methyladenosine, or rolling circle amplification 6.

Under physiological conditions, circRNAs are involved in tissue and organ growth and development, and they regulate numerous cellular processes, including the cell cycle, cell stress, cellular senescence, metabolic activity, apoptosis, and inflammatory responses. Since circRNAs are resistant to standard RNA degradation pathways, cells eliminate them primarily via exocytosis. CircRNAs or their complexes are packaged into vesicles that are secreted into the extracellular space for removal from the cytoplasm 7. Furthermore, the endoribonuclease RNase L degrades circRNAs 8.

CircRNAs accumulate primarily in slowly proliferating cells. Their expression varies across developmental stages and is altered in many diseases. Their low expression has hampered their characterization; however, because of their distinctive tissue-specific expression, they are useful as diagnostic biomarkers and novel therapeutic agents 3. Next-generation sequencing and genome mapping tools have positioned circRNAs as a key focus of RNA research.

Understanding the functions of circRNAs in liver diseases is essential, as it may facilitate the discovery and validation of novel diagnostic biomarkers and therapeutic agents for disorders with substantial global health burdens. This review aimed to critically examine the evolving functions of circRNAs in the progression and pathogenesis of liver diseases.

## CircRNA in the Liver

The liver is comprised of various cells that work together to regulate essential functions. Hepatic cells mediate lipid homeostasis, glucose metabolism, energy balance, immune responses, and detoxification. Globally, liver diseases are major causes of morbidity and impose a substantial economic burden on healthcare systems. They contribute to over two million deaths each year: one million due to complications from fibrosis and cirrhosis, and one million attributed to hepatocellular carcinoma (HCC) and viral hepatitis 9. The mechanisms driving liver disease development are not fully understood, and treatment options for end-stage liver conditions remain limited, highlighting the need for early interventions and novel therapeutic strategies.

CircRNAs are important regulators of liver homeostasis and disease. One study demonstrated that 668 circRNAs are expressed exclusively in liver tissues 10. In addition, the RAISE pipeline detected circRNAs in RNA-seq data from 61 human liver samples after rRNA depletion. In total, 59,128 circRNA candidates were observed in both adjacent non-tumor and HCC tissues 11.

## CircRNAs in Liver Diseases

### HCC

In 2020, HCC ranked sixth in global cancer incidence and third in cancer-related mortality. It accounts for 75‒85% of all primary liver cancer cases. Primary contributors to the risk of developing HCC include metabolic syndrome, viral infection, prolonged alcohol consumption, and conditions related to obesity and diabetes. Despite advances in treatments, including surgery and systemic drug therapies, the overall survival rate for patients with HCC remains poor, largely due to late diagnosis and the high degree of tumor heterogeneity in this cancer type 12.

#### Oncogenic circRNAs in HCC

circRNAs are crucial for regulating the initiation and progression of HCC 13. For example, hsa_circ_0005075 interacts with miR-93-3p, miR-23b-5p, miR-23a-5p, and miR-581 to regulate cell adhesion during HCC development 14. In hepatitis B-related HCC, upregulation of circRNA_100338, which targets miR-141-3p, is associated with poor survival and metastatic progression 15. Additional circRNAs implicated in liver cancer include circFUT8, circZFR, and circIOP11. Specifically, circFUT8 competitively binds to miR-17-3p, miR-570-3p, and miR-552-3p; circZFR potentially interacts with miR-130b-5p, miR-511-5p, miR-642a-5p, miR-329-5p, and miR-532-3p; and circIPO11 targets miR-659-3p, miR-106a-3p, and miR-424-5p 16. Circ-HOMER1, which is upregulated in HCC cells and tissues, stimulates HCC growth by targeting the miR-1322/CXCL6 axis 17. RhoA and circ_000839 levels are elevated in HCC, and miR-200b expression is negatively correlated with circ_000839 levels 18. Circβ-catenin is upregulated in liver cancer tissues, and its downregulation reduces β-catenin protein concentrations without affecting its mRNA concentration. Circβ-catenin also plays a role in stabilizing full-length β-catenin by counteracting GSK3β-mediated phosphorylation and subsequent β-catenin degradation, thereby contributing to Wnt pathway activation 19. Elevated levels of circACVR2A have been detected in HCC cell lines. CircACVR2A interacts with miR-511-5p to regulate the signaling axis 20. Both HCC tissues and cells exhibit high levels of circPIAS1, and suppression of circPIAS1 reduces cell proliferation and migration. Overexpression of circPIAS, which binds to miR-455-3p, inhibits ferroptosis and upregulates nuclear protein 1, which subsequently activates FTH1 transcription, promoting iron storage 21. In HCC, circESYT2 is upregulated and promotes tumor growth and metastasis by interacting with the miR-665/enolase 2 axis 22. Elevated levels of circ_0067934 in HCC tissues, which correlate with increased tumor metastasis and growth, are associated with the miR-1324 and Wnt/β-catenin pathway 23. In HCC, SCD-circRNA2 expression, which is regulated by RNA-binding protein 3 (RBM3), is upregulated. Modulation of SCD-circRNA2 and RBM3 levels increases HCC cell proliferation. Furthermore, RBM3-SCD-circRNA2 regulates p-ERK activation 24. Hsa_circ_0000092, which targets miRNA-338-3p, is upregulated in HCC, and its downregulation correlates with reduced cell invasion, proliferation, and angiogenesis by decreasing HN1 expression 25. Circ-PRMT5 overexpression potentially plays a critical role in HCC cell glycolysis, migration, and proliferation by targeting miR-188-5p/HK2 26. Mechanistically, circMAT2B stimulates the expression of the glycolytic enzyme PKM2 by interacting with miR-338-3p 27. Glycolysis plays a pivotal role in HCC owing to its role in metabolic reprogramming, which is a hallmark of cancer. In the presence of sufficient oxygen, HCC cells preferentially utilize aerobic glycolysis rather than mitochondrial oxidative phosphorylation to generate ATP and biosynthetic macromolecules essential for rapid proliferation. Accelerated HCC growth usually exceeds angiogenesis, resulting in hypoxic conditions that further reinforce glycolytic flux to sustain anabolic demands 26, 27. CircASAP1, which regulates miR-532-5p- and miR-326-mediated signaling, increases in HCC tissues. CircASAP1 stimulates HCC cell invasion and proliferation, and regulates macrophage infiltration into tumor tissues 28. CircUHRF1, secreted by HCC cells, suppresses the immune response by inhibiting natural killer cell function via upregulation of TIM-3 expression 29. CircRHOT1, which modulates TIP60 recruitment to the *NR2F6* promoter, contributes to the activation of *NR2F6* transcription and is upregulated in HCC 30. Circ_0000105 is overexpressed in liver cancer and enhances phosphoinositide-3-kinase regulatory subunit 1 expression by targeting miR-498; its overexpression correlates with increased HCC proliferation and reduced apoptosis 31. Circ_0091579, which targets miRNA-490-3p, is upregulated in HCC. Its downregulation strongly inhibits cell proliferation and metastasis 32. High levels of circPRKCI in HCC suppress apoptosis and promote invasion by interacting with miRNA-545 to regulate E2F7 and reduce AKT3 protein expression 33. HCC migration, proliferation, and invasion increase via the upregulation of hsa_circRNA_100084, which may stimulate insulin-like growth factor 2 (IGF2) by sequestering miR-23a-5p 34. CircSOD2 overexpression correlates with cell migration, cell growth, and the cell cycle. CircSOD2 inhibits miR-502-5p, thereby suppressing SOCS3 expression and activating the Janus kinase 2 (JAK2)/STAT3 pathway 35. MUC1, which is elevated in tumor cells, is repressed by miRNA-485-5p. Downregulation of MUC1 in cells is correlated with decreased cell viability, invasion, and migration but increased apoptosis. Upregulation of circHECTD1, which interacts with miRNA-485-5p, increases MUC1 expression and promotes HCC progression 36. In HCC, circ_0016788 is also elevated 37,38. Loss of circ_0016788 inhibits tumor growth *in vivo*; *in vitro*, HCC cell proliferation, invasion, colony formation, cell vitality, and glycolysis are suppressed, whereas apoptosis is enhanced. Circ_0016788 mediates its effects through themiR-506-3p/ poly(ADP-ribose) polymerase family member 14 (PARP14) and miR-486/CDK4 pathways 37,38. CircZNF566 is implicated in HCC metastasis and tumorigenesis; *in vitro*, it targets the tryptophan 2,3-dioxygenase axis via miR-4738-3p to promote cell invasion, proliferation, and migration 39. CircTMEM45A, which acts via the miR-665/IGF2 axis, is upregulated in HCC and correlates with tumorigenesis and the progression of cell mobility 40. Tumor stage, size, and vascular invasion are correlated with elevated circ-0046600 expression, and suppression of circ-0046600 inhibits cell migration. Most hsa-circ-0046600 is located in the cell cytoplasm, where it stimulates HIF-1α expression through binding miR-640 41. The knockdown of circMAN2B2, which is markedly elevated in HCC, suppresses cell proliferation by sponging the miR-217 and regulating the mitogen-activated protein kinase 1 pathway 42. Increased circPTGR1 expression, which has three isoforms, in serum exosomes of patients with HCC is indicative of advanced tumor stage and worse prognosis. Loss of circPTGR1 is correlated with reduced migration and invasion of 97L and HepG2 cells. The circPTGR1 isoforms and MET compete to specifically bind miR449a 43. Elevated circPVT1 expression in HCC is associated with reduced miR-377 levels. Knockdown of circPVT1 impairs HCC tumor growth, suppresses glycolysis and proliferation, and enhances apoptosis. CircPVT1 in HCC is regulated by binding to miR-377 44. Circ_0008450 overexpression has been detected in HCC 45,46. Downregulation of hsa_circ_0008450 results in reduced migration, proliferation, and invasion, while enhancing apoptosis. Hsa_circ_0008450 upregulates zeste homolog 2 by sponging miR-214-3p and miR-548p 45,46. CircRNA-104718, which is overexpressed in HCC, binds to miR-218-5p, thereby increasing the expression of thioredoxin domain-containing protein 5. Thus, higher circRNA-104718 levels accelerate invasion, proliferation, and migration, while downregulating apoptosis in HCC cells. In a mouse model, upregulation of circRNA-104718 increased tumor size and promoted HCC metastasis 47. Circ-ZNF652 is elevated in both the serum and tumor cells of patients with HCC. Circ-ZNF652 influences cell glycolysis, invasion, proliferation, and migration by interacting with miR-29a-3p, thereby modulating guanylyl cyclase domain-containing 1 48. Increased circ_0000267 expression correlates with poor prognosis and increased severity of HCC, and its upregulation stimulates cell growth by binding to miR-646 49. Circ-FOXP1 is significantly upregulated in the serum and tissues of patients with HCC. Circ-FOXP1 overexpression upregulates the oncogenic transcription factor sex-determining region Y-box 9 through miR-875-3p and miR-421. Its overexpression leads to accelerated tumor growth and reduced apoptosis in HCC cells 50. Upregulation of hsa_circ_101280 in HCC, which is associated with enhanced tumor cell proliferation and decreased apoptosis, is mediated by miR-375 sponging and JAK2 activation 51. In HCC cells, knockdown of circFBLIM1 levels inhibits invasion and proliferation while enhancing apoptosis. CircFBLIM1 sponges miR-346 to regulate FBLIM1 expression 52. In HCC cells, knocking down high circABCC2 levels reduces invasion and proliferation while enhancing apoptosis. CircABCC2 upregulates ABCC2 expression by interacting with miR-665 53.

#### Tumor-suppressor circRNAs in HCC

Lower hsa_circ_0001649 expression in HCC is associated with the presence of a tumor embolus and larger tumor size 54,55. Both* in vitro* and *in vivo* studies have demonstrated that the upregulation of circ-0001649 reduces HCC migration and proliferation. Hsa_circ_0001649 mediates its effects through activation of SNF2 histone linker PHD RING helicase by sponging miR-4688, miR-127-5p, and miR-612 54. Circ-ITCH expression is markedly reduced in cancerous tissues compared to controls 56. Downregulation of hsa_circ_0005986 accelerates HCC cell proliferation, and higher levels of hsa_circ_0005986 correlate with better survival outcomes in patients with cancer. Circ_0005986 downregulation results in increased miR-129-5p expression, which reduces Notch1 mRNA levels 57. Hsa_circ_0004018, which is suppressed in HCC relative to adjacent non-tumorous tissues, is involved in HCC metastasis and carcinogenesis via the miR-626/miR-30e-5p-MYC pathway 58. Patients with HCC and reduced circMTO1 expression (hsa_circRNA_104135/ hsa_circRNA_0007874) have shorter survival times. CircMTO1 is associated with miR-9, and its silencing in HCC decreases p21 levels, which promotes tumor growth and invasion 59. Decreased hsa-circ-0000221 expression correlates with reduced PTPN11 mRNA expression in the serum of patients with HCC. PTPN11 inhibits proliferation in various human cancers, while these patients exhibit higher miR-661 expression. Overexpression of hsa-circ-0000221 reduces cell viability and increases apoptosis, correlating with the accumulation of G1 and upregulation of CCDN1 in HCC. Hsa-circ-0000221 regulates PTPN11 expression by inhibiting miR-661 60. The zinc-finger family gene *ZKSCAN1* and its circRNA, circZKSCAN, are downregulated in HCC. Inhibition of circZKSCAN1 induces cell proliferation, invasion, and migration. CircZKSCAN1 modulates cancer-associated pathways, including integrin β4, transforming growth factor-β1 (TGF-β1), and chemokine receptor 4 61. Hsa_circ_0067531, which is critical for HCC development via phosphoinositide 3-kinase, is reduced in human HCC tissues *in vitro* 62. In HCC, circTRIM33-12 expression is reduced and correlates with increased invasion, migration, proliferation, and immune evasion. CircTRIM33-12 promotes Ten-eleven translocation methylcytosine dioxygenase 1 expression and reduces 5-hydroxymethylcytosine levels through miR-191 63. CircHIAT1 suppresses tumor growth by acting as a sponge for miR-3171, thereby upregulating PTEN, and is downregulated in patients with HCC 64. CircLARP4 is also downregulated in HCC. CircLARP, which mediates cell cycle arrest *in vitro*, reduces HCC proliferation and promotes senescence. HCC progression is inhibited by circLARP through miR-761, thereby activating the RUNX3 and p53/p21 pathway 65. The altered expression of circRNAs and their implicated roles in HCC are summarized in **Table 1.**

### Nonalcoholic fatty liver disease (NAFLD) and nonalcoholic steatohepatitis (NASH)

NAFLD, the primary cause of chronic liver disease worldwide, is characterized by abnormal fat accumulation in hepatocytes. Triglyceride accumulation, lipid peroxidation, and mitochondrial dysfunction are the hallmarks of liver steatosis 66. With the global prevalence of NAFLD at approximately 30%, it may increase over the next two decades. Therefore, raising awareness and promoting the understanding of NAFLD remain crucial. The inadequate response of healthcare organizations places a substantial strain on healthcare systems and economies 66.

NAFLD is a multisystem condition associated with metabolic disorders. NASH, a more advanced form of NAFLD, can progress to hepatic fibrosis, cirrhosis, and eventually liver cancer. The hallmark features of NASH include hepatocyte ballooning and hepatic inflammation 66. NAFLD affects both children and adults, with its prevalence increasing with age in males between 45 and 65 years 67. Genetic factors, including polymorphisms in *PNPLA3* and *TM6SF2* genes, elevate the risk of NAFLD 68.

CircRNAs are increasingly recognized for their distinctive expression patterns in NAFLD. Significant differences in circRNA expression have been observed in NASH and NAFLD animal models 69. In one NAFLD animal model, 231 circRNAs were upregulated and 165 were downregulated, as quantified using a circRNA microarray 70. Another study revealed that the expression of 57 circRNAs was upregulated and that of 36 circRNAs was downregulated in a high-fat diet-fed mouse model. CircRNAs also regulate the expression of *DDAH1* and *VAV3* genes in NAFLD 71.

Circ_0057558 levels were elevated in NAFLD models. This circRNA acts as a sponge for miR-206 and enhances triglyceride production and lipogenesis by relieving the repression of AMP-activated protein kinase (AMPK) and Rho-kinase 1 signaling pathways 72. CircRNA_002581 is also highly expressed in NASH. Knockdown of circRNA_002581 reduces hepatic inflammation, oxidative stress, and lipid accumulation, whereas its overexpression alleviates the suppression of cytoplasmic polyadenylation element-binding protein by miR-122. CircRNA_002581 contributes to NASH pathogenesis by suppressing autophagy through the PTEN-AMPK-mTOR axis 73. CircRNA-homeodomain-interacting protein kinase 3 is induced in patients with NASH compared to controls, suggesting that its interaction with miRNA-29a regulates NASH pathogenesis. miRNA-29a may contribute to NASH pathogenesis by decreasing the activity of the Wnt-β-catenin pathway 74.

CircRNA_0046367 expression is diminished in hepatocellular steatosis models; however, its restoration interferes with miR-34a suppression of peroxisome proliferator-activated receptor α (PPARα) 75. Restoration of PPARα activity improves hepatocellular steatosis by modulating genes involved in fatty acid oxidation, transport, and lipid metabolism 75. CircRNA_0046366 plays a critical role in lipid metabolism, as its levels decrease in free fatty acid (FFA)-induced hepatocellular steatosis. CircRNA_0046366 counteracts the inhibitory impacts of miR-34a on PPARα. Enhanced gene expression of triglyceride-specific lipolysis, such as solute carrier family 27A, correlates with restored PPARα expression, thereby decreasing triglyceride content and alleviating hepatocellular steatosis 76. CircScd1 expression is reduced in NAFLD tissues and regulates the degree of lipid accumulation. CircScd1 also reduces steatosis by activating the JAK2/STAT5 pathway 77. In addition, hsa_circ_0048179 levels are suppressed in NAFLD models induced by oleate and palmitate, along with decreased glutathione peroxidase 4 (GPX4) expression, an antioxidant enzyme that protects cells by preventing peroxidation of membrane lipids. Overexpression of Hsa_circ_0048179 stimulates GPX4 expression via miR-188-3p, attenuating oleate/palmitate-induced reactive oxygen species (ROS), lipid accumulation, steatosis, and mitochondrial dysfunction in HepG2 cells 78. In patients with NASH, the mitochondrial steatohepatitis-associated circRNA ATP5B Regulator (SCAR) limits fibroblast activation and mitochondrial ROS production. A reduction in its expression correlates with the transition from steatosis to NASH and insulin resistance. *In vivo*, modulation of circRNA SCAR attenuates insulin resistance and cirrhosis associated with a high-fat diet 79. In rodents with NAFLD, a high-fat, high-cholesterol diet disrupts the hepatic circRNA profile, notably reducing 28 circRNAs. LNCPINT-derived circRNAs are critical regulators of circRNA, miRNA, and mRNA interactions. Deficiency of these circRNAs relieves the inhibition of miR-669c-3p and miR-466i-3p, resulting in AMPK deactivation. Downregulation of the AMPK pathway promotes lipogenic gene expression and drives hepatic steatosis 80. CircRNA_0001805 expression is decreased in high-fat diet-fed mice, FFA-treated hepatocytes, and patients with NAFLD. Enhanced expression of circRNA_0001805 correlates with the attenuation of lipid metabolism abnormalities and inflammatory activity. CircRNA_0001805 targets miR-106a-5p and miR-320a, both of which act as upstream suppressors of ABCA1/CPT1 81. In hepatic steatosis, circRNA_021412 modulates the miR-1972/LPIN1 pathway, which regulates the expression of steatosis-related genes through PPARα activation 82. **Table 2** presents the correlation between circRNAs and NAFLD.

### Hepatic Fibrosis

Liver fibrosis results from excessive buildup of extracellular matrix components triggered by ongoing liver damage, which promotes wound healing. Fibrosis-related liver injuries result from various factors, including chronic hepatitis B or C infections, drug use, genetic conditions, excessive alcohol intake, metabolic disorders, autoimmune diseases, and cholestasis. The rising incidence of type 2 diabetes has led to an increase in liver fibrosis due to NASH. Liver fibrosis can be reversed at early stages if the underlying causes are addressed. However, progression of fibrosis can cause cirrhosis, liver failure, portal hypertension, and HCC 83. Hepatic fibrosis involves various cell types and mediators. Among these, hepatic stellate cells (HSCs), which are considered the main producers of fibrous matrices, are crucial for the initiation and progression of liver fibrosis and serve as primary effector cells during fibrosis development. Following liver injury, quiescent HSCs are activated by cytokines—such as interleukin 6 (IL-6), IL-17, and IL-22—which are secreted by adjacent cells. Once activated, HSCs transdifferentiate into myofibroblasts, which are involved in fibrosis development 84. HSC activation is also triggered by lipopolysaccharide (LPS) and TGF-β, both of which increase the production of fibrotic markers 84. Excessive production of the extracellular matrix disrupts the liver's structure, leading to the replacement of hepatocytes with scar tissue. Examples of extracellular matrix include fibronectin, laminin, collagen I, and collagen III. Since many circRNAs are involved in advancing or suppressing hepatic fibrosis, they represent promising biological markers for tracking the progression of liver fibrosis. In addition, these circRNAs provide critical insights into the mechanisms underlying hepatic fibrosis and highlight promising targets for diagnostic and therapeutic applications 84.

In irradiated HSCs, 179 circRNAs were highly expressed, whereas 630 circRNAs showed reduced expression relative to normal conditions. Among these altered circRNAs, hsa_circ_0072765, hsa_circ_0054345, and hsa_circ_0071410 were significantly upregulated, whereas hsa_circ_0070963, hsa_circ_0013255, and hsa_circ_0061893 were significantly downregulated. Silencing hsa_circ_0071410 elevated miR-9-5p expression, which attenuated HSC activation. Downregulation of miR-9-5p mitigates the effect of hsa_circ_0071410 inhibition, thereby reducing HSC activation 85. CircUbe2k is upregulated in mice treated with carbon tetrachloride (CCl_4)_ to induce liver fibrosis, as well as in LX-2 cells stimulated with TGF-β1. CircUbe2k increases TGF-β2 activity by targeting miR-149-5p, and suppression of circUbe2k inhibits the expression of fibrotic markers such as alpha smooth muscle actin (α-SMA) and collagen, type I, alpha 1** (**Col1α1) 86. Circ-PWWP2A expression promotes HSC activation and proliferation after LPS and TGF-β treatment, with MiR-223 and miR-203 identified as its downstream targets 87. CircRNA-0067835 is markedly upregulated in LX-2 cells lacking thymosin β4, and silencing of this circRNA suppresses cell proliferation and enhances apoptosis. Bioinformatic analyses predict that circRNA-0067835 interacts with miR-155, thereby modulating forkhead box O3 expression through its sponging activity 88. In LX2 cells exposed to radiation, CircRSF1 expression is increased and predicted to bind to miR-146a-5p. The circRSF1-miR-146a-5p complex increases cell viability, fibrosis, and inflammation via RAC1 activation 89. CircTUBD1 promotes liver fibrosis by modulating miRNA-203a-3p and Smad signaling. In a radiation-induced liver fibrosis model, reducing circTUBD1 activity suppresses hepatic fibrosis biomarkers 90. Hsa_circ_0072765 is upregulated in HSCs exposed to TGF-β1, and its knockdown ameliorates HSC activation and migration through the transient receptor potential vanillin 3 pathway 91. In CCl_4_-treated mice and RAW264.7 cells stimulated with IFN-γ and LPS, circMcph1 expression is elevated. CircMcph1 regulates IL-1 receptor-associated kinase 2 (Irak2) activity during liver fibrosis by sponging miR-370-3p 92. CircRNA-007371 exhibits angiogenic effects in a mouse fibrosis model induced by thioacetamide. Overexpression of circRNA-007371 promotes cell proliferation. Acting as a miRNA sponge, circRNA-007371 enhances angiogenesis 93. CircRNA cVIM facilitates the upregulation of TGF-β receptor subtypes through miR-122-5p and miR-9-5p, resulting in the activation of the TGF-β/Smad signaling axis 94. Circ_0008494 functions as a sponge for miR-185-3p in human fibrotic tissues, and knockdown of this interaction reduces HSC proliferation, activation, and migration while promoting apoptosis 95.

CircPSD3, derived from the pleckstrin and Sec7 domain-containing 3 (*PSD3*) gene, is reduced in CCl_4_-treated mouse livers and primary HSCs. Furthermore, overexpression of circPSD3 is associated with decreased collagen deposition, reduced liver enzyme and hydroxyproline levels, and lower expression of profibrogenic and proinflammatory cytokines. CircPSD3 acts as a miR-92b-3p sponge, enhancing Smad7 expression 96. CircFBXW4 expression is reduced during liver fibrosis. Increased circFBXW4 suppresses HSC proliferation and activation, promotes apoptosis, mitigates hepatic fibrotic damage in mice, and exhibits anti-inflammatory properties. Mechanistically, circFBXW4 interacts with miR-18b-3p during hepatic fibrosis 97. Hsa_circ_0004018 expression decreases during liver fibrogenesis, and its overexpression suppresses fibrosis progression. By sponging hsa-miR-660-3p, hsa_circ_0004018 may indirectly reduce TEP1 expression 98. CircCREBBP expression is also reduced during liver fibrosis. Overexpression of circCREBBP prevents liver fibrosis progression by inhibiting HSC proliferation and activation. Mechanistically, circCREBBP enhances LEFTY2 expression by interacting with hsa-miR-1291 99. Hsa_circ_0070963 reduces fibrotic damage by modulating miR-223-3p, which interacts with LEMD3. This circRNA is downregulated during fibrosis; however, restoring its normal expression suppresses HSC activation and attenuates fibrotic biomarkers 100. *In silico* analysis indicated that the regulatory role of mmu_circ_34116 in HSC activation was mediated through the miR-22-3P/BMP7 signaling pathway. Suppression of mmu_circ_34116 stimulates α-SMA expression 101. CircDIDO1 inhibits fibrosis by acting as an miR-141-3p sponge. CircDIDO1 overexpression downregulates profibrotic markers, suppresses proliferation, and enhances apoptosis and cell cycle arrest in HSCs by suppressing the PTEN/AKT pathway 102. Hsa_circ_0007874 (circMTO1), derived from the *MTO1* gene, is downregulated in fibrotic mouse livers and activated HSCs. Enhancing circMTO1 expression inhibits the activation of HSCs triggered by TGF-β1. CircMTO1 targets PTEN and Smad7 via miR-17-5p and miR-181b-5p 103,104.** Table 3** summarizes the circRNAs associated with the regulation of liver fibrosis.

## Conclusions

This review highlights the biogenesis, function, and importance of circRNAs, emphasizing their essential roles as hallmarks of HCC, NAFLD, NASH, and liver fibrosis. However, further research is needed to elucidate the mechanisms underlying their synthesis, biological functions, and clearance. The effects of circRNAs on liver disease through their interactions with miRNAs indicate their potential as biomarkers for prognosis, diagnosis, and targeted therapy. Despite this promise, translating the mechanisms identified by basic research into clinical applications for early detection and prognosis of liver disease remains a considerable challenge. Understanding the dysregulation of circRNA expression in liver diseases may clarify their roles in regulating various molecules and signaling pathways. Nonetheless, their contributions to liver homeostasis, disease development, and the molecular mechanisms regulating circRNAs remain poorly understood. The distinct functions of various liver cell types in liver pathogenesis complicate efforts to determine the precise roles of circRNAs in liver disease. In addition, studies linking circRNAs with liver diseases have focused primarily on HCC, highlighting the need to expand investigations to other liver diseases. Finally, the regulatory effects of circRNA polymorphisms on circRNA expression and function remain to be elucidated.

## Figures and Tables

**Table 1:** Expression and roles of dysregulated circular RNAs in hepatocellular carcinoma

Reference	Biological Function	Sponge Target	Expression Pattern	Circular RNA
14	Hsa_circ_0005075 regulates cell adhesion.	miR-93-3p miR-23b-5p miR-23a-5p miR-581	Increased	Hsa_circ_0005075
15	Elevated circRNA_100338 levels are linked to decreased survival and metastatic advancement.	miR-141-3p	Increased	CircRNA_100338
16	Not identified	miR-570-3p miR-17-3p miR-552-3p	Increased	CircFUT8 Hsa_circ_0003028 Hsa_circ_101368
16	Not identified.	miR-511-5p miR-532-3p miR-130b-5p miR-329-5p miR-642a-5p	Increased	CircZFR Hsa_circ_10072088 Hsa_circRNA_103809
16	Not identified.	miR-659-3p miR-106a-3p miR-424-5p	Increased	CircIOP11 Hsa_circ_0007915 Hsa_circ_103847
17	Circ-HOMER1 stimulates migration, proliferation, and invasion, while reducing apoptosis. HOMER1 targets CXCL6.	miR-1322	Increased	Circ-HOMER1
18	Not identified.	Not identified	Increased	Circ_000839
19	Circβ-catenin activates Wnt/β-catenin.	Not identified	Increased	Circβ-catenin
20	CircACVR2A regulates invasion, proliferation, and migration. CircACVR2A regulates PI3K-Akt.	miR-511-5p	Increased	CircACVR2A
21	CircPIAS1 knockdown reduces migration and proliferation. Its overexpression suppresses ferroptosis and upregulates NUPR1.	miR-455-3p	Increased	CircPIAS1
22	CircESYT2 mediates metastasis and growth through its interaction with ENO2.	miR-665	Increased	CircESYT2
23	Circ_0067934 overexpression promotes growth and metastasis. It regulates FZD5/Wnt/β-catenin.	miR-1324	Increased	Circ_0067934
24	SCD-circRNA2 upregulation is mediated by RBM3. RBM3-SCD-circRNA2 promotes proliferation and p-ERK activation.	Not identified	Increased	SCD-circRNA2
25	Hsa_circ_0000092 increases progression by upregulating HN1 expression.	miR-338-3p	Increased	Hsa_circ_0000092
26	Circ-PRMT5 mediates migration, proliferation, and glycolysis through the HK2 axis.	miR-188-5p	Increased	Circ-PRMT5
27	CircMAT2B stimulates *PKM2* gene expression, which encodes a glycolysis enzyme.	miR-338-3p	Increased	CircMAT2B
28	CircASAP1 stimulates invasion and proliferation and regulates the infiltration of macrophages.	miR-326 miR-532-5p	Increased	CircASAP1
29	CircUHRF1 regulates immunosuppression by inhibiting natural killer cell functions through upregulation of TIM-3 expression.	Not identified	Increased	CircUHRF1
30	CircRHOT1 promotes HCC proliferation and metastasis. It recruits TIP60 to the promoter of *NR2F6* to initiate the transcription of *NR2F6*.	Not identified	Increased	CircRHOT1
31	Circ_0000105 promotes proliferation and reduces apoptosis in HCC. Circ_0000105 promotes PIK3R1 expression.	miR-498	Increased	Circ_0000105
32	Circ_0091579 promotes HCC proliferation and metastasis.	miRNA-490-3p	Increased	Circ_0091579
33	circ-PRKCI significantly inhibits HCC cell apoptosis and promotes cell invasion.	miRNA-545	Increased	Circ-PRKCI
34	Hsa_circRNA_100084 stimulates IGF2.	miR-23a-5p	Increased	Hsa_circRNA_100084
35	CircSOD2 promotes growth, cycle, and migration. Increased DNMT3a downregulates SOCS3 and enhances JAK2/STAT3.	miR-502-5p	Increased	CircSOD2
36	CircHECTD1 facilitates MUC1 expression and promotes progression.	miRNA-485-5p	Increased	CircHECTD1
37,38	Hsa_circ_0016788 mediates CDK4 and PARP14.	miR-506-3p miR-486	Increased	Hsa_circ_0016788
39	CircZNF566 upregulation increases tumorigenesis, metastasis, invasion, migration, and proliferation. CircZNF566 regulates the TDO2 axis.	miR-4738-3p	Increased	CircZNF566
40	CircTMEM45A regulates cell mobility and tumorigenesis. Its action is mediated by the IGF2 axis.	miR-665	Increased	CircTMEM45A
41	Hsa-circ-0046600 mediates migration.	miR-640	Increased	Hsa-circ-0046600
42	CircMAN2B2 regulates proliferation via MAPK1.	miR-217	Increased	CircMAN2B2
43	CircPTGR1 isoforms stimulate migration by targeting MET.	miR449a	Increased	CircPTGR1
44	CircPVT1 regulates proliferation, glycolysis, and apoptosis by targeting TRIM23.	miR-377	Increased	CircPVT1
45,46	Hsa_circ_0008450 regulates proliferation, invasion, and apoptosis by promoting EZH2.	miR-548p miR-214-3p	Increased	Hsa_circ_0008450
47	CircRNA-104718 overexpression increases tumor size, promotes metastasis, migration, invasion, and proliferation, and inhibits apoptosis.	miR-218-5p	Increased	CircRNA-104718
48	Circ-ZNF652 regulates glycolysis, proliferation, invasion, and migration through GUCD1.	miR-29a-3p	Increased	Circ-ZNF652
49	Circ_0000267 upregulation promotes invasion, migration, and proliferation, and ameliorates apoptosis.	miR-646	Increased	Circ_0000267
50	Overexpression of circ-FOXP1 increases oncogenic transcription factor SOX9 expression, cell growth, and invasion, and suppresses apoptosis.	miR-421 miR-875-3p	Increased	Circ-FOXP1
51	Hsa_circ_101280 mediates tumorigenesis and proliferation, while reducing apoptosis by JAK2.	miR-375	Increased	Hsa_circ_101280
52	CircFBLIM1 knockdown increases apoptosis and decreases cell proliferation and invasion.	miR-346	Increased	CircFBLIM1
53	CircABCC2 inhibition reduces proliferation and invasion and increases apoptosis by regulating ABCC2.	miR-665	Increased	CircABCC2
54,55	Circ-0001649 upregulation reduces proliferation and migration. It mediates SHPRH activation.	miR-127-5p miR-4688 miR-612	Decreased	Hsa_circ_0001649
56	Polymorphisms (rs4911154 and rs10485505) in circ-ITCH increase the risk of HCC.	Not identified	Decreased	Circ-ITCH
57	Downregulation of hsa_circ_0005986 stimulates proliferation and reduces Notch1 expression.	miR-129-5p	Decreased	Hsa_circ_0005986
58	Hsa_circ_0004018 regulates metastasis and carcinogenesis by targeting MYC.	miR-626 miR-30e-5p	Decreased	Hsa_circ_0004018
59	CircMTO1 silencing reduces p21 expression as well as proliferation and invasion.	miR-9	Decreased	CircMTO1 Hsa_circRNA_104135 Hsa_circRNA_0007874
60	Low hsa-circ-0000221 expression reduces PTPN11 expression. Its overexpression lowers cell viability, increases apoptosis, and upregulates G1 protein and CCDN1.	miR-661	Decreased	Hsa-circ-0000221
61	Silencing circZKSCAN1 in HCC induces proliferation, invasion, and migration. CircZKSCAN1 influences TGF-β1, ITGB4, and CXCR4 expression.	Not identified	Decreased	CircZKSCAN1
62	Hsa_circ_0067531 regulates the PI3K pathway.	Not identified	Decreased	Hsa_circ_0067531
63	CircTRIM33-12 reduction increases migration, invasion, proliferation, and immune evasion by promoting TET1 expression and reducing 5hmC expression.	miR-191	Decreased	CircTRIM33-12
64	CircHIAT1 inhibits growth and upregulates PTEN expression.	miR-3171	Decreased	CircHIAT1
65	CircLARP4 reduces proliferation, mediates cell cycle arrest, and promotes senescence by increasing RUNX3 expression and activating p53/p21.	miR-761	Decreased	CircLARP4

**Abbreviations**: HCC: hepatocellular carcinoma; circRNA: circular RNA; miR / miRNA: microRNA; MAPK1: mitogen-activated protein kinase 1; JAK2: Janus kinase 2; EZH2: enhancer of zeste homolog 2; TXNDC5: thioredoxin domain-containing protein 5; MET: mesenchymal-epithelial transition factor; PTPN11: protein tyrosine phosphatase non-receptor type 11; CCDN1: cyclin D1; SOX9: SRY-box 9; FZD5: frizzled class receptor 5; PKM2: pyruvate kinase M2; HKL2: hexokinase-like 2; DNMT3a: DNA methyltransferase 3 alpha; SOCS3: suppressor of cytokine signaling 3; STAT3: signal transducer and activator of transcription 3; IGF2: insulin-like growth factor 2; CDK4: cyclin-dependent kinase 4; PARP14: poly (ADP-ribose) polymerase family member 14; TDO2: tryptophan 2,3-dioxygenase; GUCD1: guanylyl cyclase domain containing 1; SHPRH: SNF2 histone linker PHD RING helicase; ITGB4: integrin beta 4; TGF-β1: transforming growth factor beta 1; CXCR4: chemokine receptor 4; PI3K: phosphoinositide 3-kinase; TET1: ten-eleven translocation methylcytosine dioxygenase 1; 5hmC: 5-hydroxymethylcytosine; PTEN: phosphatase and tensin homolog; RUNX3: runt-related transcription factor 3; p-ERK: phosphorylated extracellular signal-regulated kinase; TRIM23: tripartite motif containing 23; FBLIM1: filamin-binding LIM protein 1.

**Table 2:** Expression and roles of dysregulated circular RNAs in nonalcoholic fatty liver disease

Reference	Biological Function	Sponge Target	Expression Pattern	Circular RNA
72	Circ_0057558 promotes lipogenesis and triglyceride secretion by relieving the repression of ROCK1 and AMPK.	miR-206	Increased	Circ_0057558
73	CircRNA_002581 regulates CPEB1. CircRNA_002581 knockdown reduces oxidative stress, lipid accumulation, and inflammation.	miR-122	Increased	CircRNA_002581
74	CircRNA-HIPK3 overexpression downregulates Wnt/β-catenin, contributing to NASH pathogenesis.	miRNA-29a	Increased	CircRNA-HIPK3
75	Normal circRNA_0046367 concentrations abolish the inhibitory effects of PPARα.	miR-34a	Decreased	CircRNA_0046367
76	CircRNA_0046366 reduces triglycerides and ameliorates hepatocellular steatosis. Normal circRNA_0046366 concentrations abolish the inhibition of PPARα.	miR-34a	Decreased	CircRNA_0046366
77	CircScd1 reduces the severity of lipid accumulation by JAK2/STAT5 activation.	Not identified	Decreased	CircScd1
78	Hsa_circ_0048179 accelerates GPX4 and reduces reactive oxygen species and lipid accumulation.	miR-188-3p	Decreased	Hsa_circ_0048179
79	CircRNA SCAR suppresses reactive oxygen species and fibroblast activation. It binds to ATP5B and suppresses mPTP activity by preventing CypD and mPTP linkage.	ATP5B	Decreased	CircRNA SCAR
80	Circ_0001452 suppression inhibits AMPK and promotes lipogenic gene expression.	miR-669c-3p miR-466i-3p	Decreased	Circ_0001452 Circ_0001453 Circ_0001454
81	CircRNA_0001805 overexpression decreases lipid accumulation and mitigates lipid metabolism disorders and inflammation. It acts as ABCA1/CPT1 suppressor	miR-106a-5p miR-320a	Decreased	CircRNA_0001805
82	CircRNA_021412 regulates LPIN1, inducing steatosis-related genes via PPARα activation.	miR-1972	Decreased	CircRNA_021412

**Abbreviations:** AMPK: AMP-activated protein kinase; ROCK1: Rho-kinase 1; CPEB1: cytoplasmic polyadenylation element-binding protein 1; NASH: nonalcoholic steatohepatitis; PPARα: peroxisome proliferator-activated receptor alpha; JAK2: Janus kinase 2; STAT5: signal transducer and activator of transcription 5; GPX4: glutathione peroxidase 4; ATP5B: ATP synthase subunit beta; mPTP: mitochondrial permeability transition pore; CypD: cyclophilin D; ABCA1: ATP-binding cassette transporter A1; CPT1: carnitine palmitoyltransferase 1; miR / miRNA: microRNA.

**Table 3:** Expression and roles of dysregulated circular RNAs in liver fibrosis

Reference	Biological Function	Sponge Target	Expression Pattern	Circular RNA
85	Hsa_circ_0071410 downregulation reduces HSCs activation.	miR-9-5p	Increased	Hsa_circ_0071410
86	CircUbe2k increases TGF-β2 activity, and its suppression reduces α-SMA and Col1α1.	miR-149-5p	Increased	CircUbe2k
87	Circ-PWWP2A regulates HSC activation and proliferation.	miR-223 miR-203	Increased	Circ-PWWP2A
88	CircRNA-0067835 regulates fibrosis progression by modulating FOXO3a expression. Its silencing suppresses HSC proliferation and enhances apoptosis.	miR-155	Increased	CircRNA-0067835
89	CircRSF1 increases cell viability, cell fibrosis, and inflammation by activating RAC1.	miR-146a-5p	Increased	CircRSF1
90	CircTUBD1 induces liver fibrosis by regulating Smad3. A reduction in circTUBD1 suppresses the expression of COL1A1, COL3A1, and CTGF.	miR-203a-3p	Increased	CircTUBD1
91	Hsa_circ_0072765 suppression inhibits activation, migration, and proliferation by targeting TRPV3.	miR-197-3p	Increased	Hsa_circ_0072765
92	A reduction in circMcph1 decreases fibrosis and inflammation. It modulates Irak2 expression.	miR-370-3p	Increased	CircMcph1
93	CircRNA-007371 overexpression increases angiogenesis, proliferation, and migration.	Not identified	Increased	CircRNA-007371
94	CircRNA cVIM activates HSCs and regulates TGFBR, resulting in TGF-β/Smad activation.	miR-9-5p miR-122-5p	Increased	CircRNA cVIM
95	Circ_0008494 downregulation decreases HSC proliferation, activation, and migration and stimulates apoptosis. Col1a1 is its target.	miR-185-3p	Increased	Circ_0008494
96	CircPSD3 overexpression decreases collagen, profibrogenic and proinflammatory markers, liver enzymes, and liver hydroxyproline by enhancing Smad7.	miR-92b-3p	Decreased	CircPSD3
97	CircFBXW4 suppresses hepatic inflammation, HSC proliferation, and activation, and promotes apoptosis by controlling FBXW7.	miR-18b-3p	Decreased	CircFBXW4
98	Hsa_circ_0004018 suppresses fibrosis by inhibiting TEP1.	miR-660-3p	Decreased	Hsa_circ_0004018
99	CircCREBBP reduces the activation and proliferation of HSCs. It is involved in LEFTY2 expression.	miR-1291	Decreased	CircCREBBP
100	Hsa_circ_0070963 reduces HSC activation and α-SMA and type I collagen expression. Its antifibrotic effect is mediated by LEMD3 regulation.	miR-223-3p	Decreased	Hsa_circ_0070963
101	Mmu_circ_34116 regulates HSC activation. Its inhibition is associated with increased α-SMA expression.	miR-22-3P	Decreased	Mmu_circ_34116
102	CircDIDO1 decreases profibrotic markers and HSC proliferation and induces apoptosis by inhibiting the PTEN/AKT pathway.	miR-141-3p	Decreased	CircDIDO1
103,104	CircMTO1 decreases HSC proliferation as well as the expression of α-SMA and type I collagen by PTEN and Smad7 regulation.	miR-17-5p miR-181b-5p	Decreased	CircMTO1

**Abbreviations:** HSCs: hepatic stellate cells; TGF-β1: transforming growth factor beta 1; α-SMA: alpha-smooth muscle actin; Col1α1 / COL1A1: collagen type I alpha 1 chain; COL3A1: collagen type III alpha 1 chain; CTGF: connective tissue growth factor; TRPV3: transient receptor potential vanilloid 3; Irak2: interleukin-1 receptor-associated kinase 2; TGFBR: transforming growth factor beta receptor; Smad3 / Smad7: mothers against decapentaplegic homolog 3 / 7; FBXW7: F-box and WD repeat domain containing 7; TEP1: telomerase-associated protein 1; LEFTY2: left-right determination factor 2; LEMD3: LEM domain containing 3; PTEN: phosphatase and tensin homolog; AKT: protein kinase B; miR / miRNA: microRNA

## Abbreviations

miRNA: microRNA
circRNAs: Circular RNAs
HCC: hepatocellular carcinoma
NAFLD: nonalcoholic fatty liver diseases
Mbl: muscle-blind
NUPR1: nuclear protein 1
ENO2: enolase 2
MAPK1: mitogen-activated protein kinase 1
EZH2: zeste homolog 2
TXNDC5: thioredoxin domain-containing protein 5
GUCD1: guanylyl cyclase domain containing 1
SOX9: SRY (sex determining region Y)-box 9
JAK2: Janus kinase 2
ITGB4: integrin β4
TGF-β1: transforming growth factor-β1
CXCR4: chemokine receptor 4
SHPRH: SNF2 histone linker PHD RING helicase
PI3K: phosphoinositide 3-kinase
TET1: ten-eleven translocation methylcytosine dioxygenase 1
5hmC: 5-hydroxymethylcytosine
NASH: nonalcoholic steatohepatitis
ROCK1: Rho-kinase 1
AMPK: AMP-activated protein kinase
CPEB: cytoplasmic polyadenylation element-binding protein
HIPK3: homeodomain interacting protein kinase 3
PPARα: peroxisome proliferator-activated receptor α
FFA: free fatty acid
SLC27A: solute carrier family 27A
GPX4: glutathione peroxidase 4
ROS: reactive oxygen species
SCAR: circRNA ATP5B regulator
mROS: mitochondrial ROS
HSCs: hepatic stellate cells
IL: interleukin
LPS: lipopolysaccharide
α-SMA: alpha smooth muscle actin
Col1α1: collagen, type I, alpha 1
Tβ4: thymosin β4
FOXO3a: forkhead box O3
TRPV3: transient receptor potential vanillin 3
IRAK2: IL-1 receptor-associated kinase 2
TAA: thioacetamide
PSD3: pleckstrin and Sec7 domain-containing 3
